# Enhanced lipid production by *Yarrowia lipolytica* cultured with synthetic and waste-derived high-content volatile fatty acids under alkaline conditions

**DOI:** 10.1186/s13068-019-1645-y

**Published:** 2020-01-06

**Authors:** Ruiling Gao, Zifu Li, Xiaoqin Zhou, Wenjun Bao, Shikun Cheng, Lei Zheng

**Affiliations:** School of Energy and Environmental Engineering, Beijing Key Laboratory of Resource-oriented Treatment of Industrial Pollutants, University of Science and Technology Beijing, Beijing, 100083 People’s Republic of China

**Keywords:** Microbial lipids, *Yarrowia lipolytica*, High-content volatile fatty acids, Alkaline conditions

## Abstract

**Background:**

Volatile fatty acids (VFAs) can be effective and promising alternate carbon sources for microbial lipid production by a few oleaginous yeasts. However, the severe inhibitory effect of high-content (> 10 g/L) VFAs on these yeasts has impeded the production of high lipid yields and their large-scale application. Slightly acidic conditions have been commonly adopted because they have been considered favorable to oleaginous yeast cultivation. However, the acidic pH environment further aggravates this inhibition because VFAs appear largely in an undissociated form under this condition. Alkaline conditions likely alleviate the severe inhibition of high-content VFAs by significantly increasing the dissociation degree of VFAs. This hypothesis should be verified through a systematic research.

**Results:**

The combined effects of high acetic acid concentrations and alkaline conditions on VFA utilization, cell growth, and lipid accumulation of *Yarrowia lipolytica* were systematically investigated through batch cultures of *Y. lipolytica* by using high concentrations (30–110 g/L) of acetic acid as a carbon source at an initial pH ranging from 6 to 10. An initial pH of 8 was determined as optimal. The highest biomass and lipid production (37.14 and 10.11 g/L) were obtained with 70 g/L acetic acid, whereas cultures with > 70 g/L acetic acid had decreased biomass and lipid yield due to excessive anion accumulation. Feasibilities on high-content propionic acid, butyric acid, and mixed VFAs were compared and evaluated. Results indicated that *Y*_X/S_ and *Y*_L/S_ of cultures on butyric acid (0.570, 0.144) were comparable with those on acetic acid (0.578, 0.160) under alkaline conditions. The performance on propionic acid was much inferior to that on other acids. Mixed VFAs were more beneficial to fast adaptation and lipid production than single types of VFA. Furthermore, cultures on food waste (FW) and fruit and vegetable waste (FVW) fermentate were carried out and lipid production was effectively improved under this alkaline condition. The highest biomass and lipid production on FW fermentate reached 14.65 g/L (*Y*_X/S_: 0.414) and 3.20 g/L (*Y*_L/S_: 0.091) with a lipid content of 21.86%, respectively. By comparison, the highest biomass and lipid production on FVW fermentate were 11.84 g/L (*Y*_X/S_: 0.534) and 3.08 g/L (*Y*_L/S_: 0.139), respectively, with a lipid content of 26.02%.

**Conclusions:**

This study assumed and verified that alkaline conditions (optimal pH 8) could effectively alleviate the lethal effect of high-content VFA on *Y. lipolytica* and significantly improve biomass and lipid production. These results could provide a new cultivation strategy to achieve simple utilizations of high-content VFAs and increase lipid production. Feasibilities on FW and FVW-derived VFAs were evaluated, and meaningful information was provided for practical applications.

## Background

Lipids synthesized by oleaginous yeasts are highly promising feedstock for biodiesel production primarily because of their high potential productivity and short production period [1]. However, their large-scale application is limited by their high production costs, which mainly stem from traditional but expensive fermentation substrates, such as glucose [2]. Therefore, the exploitation of low-cost alternative substrates is becoming a research hot spot. Volatile fatty acids (VFAs), which refer to short chain fatty acids (C1–C6), can be cheaply produced from the anaerobic fermentation of various organic wastes [3]. Economic calculation results have shown that VFAs produced from food waste (FW) cost 30 dollars/ton, which is < 10% of the cost of a ton of glucose [4]. Besides their sufficient and cost-effective sources, VFAs also have higher theoretical conversion efficiencies and shorter metabolic pathways to lipids compared with other sugar-based carbon sources [5–7]. Therefore, VFAs have been considered as promising alternate carbon source for microbial lipids production. Recently, research on the bioconversion of VFAs into high value-added microbial lipids have attracted significant attention and some oleaginous yeast and very few molds have been proven to utilize VFAs to synthesize lipids, e.g. *Yarrowia lipolytica, Cryptococcus curvatus, Cryptococcus albidus* [8].

VFAs can be routinely generated during the acidogenic fermentation of various organic wastes. In general, VFAs derived from sewage excess sludge have a concentration of 2–8 g/L [9], whereas VFAs derived from FW, animal or human feces, and high-content organic wastewater can have a relatively high concentration of 10–40 g/L [10]. VFA composition can be affected by substrate type and fermentation conditions [11]. Among all types of VFAs, acetic acid is generally the highest, accounting for 43–69% [12], followed by propionic acid (10–54%) [13] and butyric acid (9–46.9%) [8]. In recent years, these waste-derived VFAs turn out to be effective carbon sources for the synthesis of high value-added microbial lipids by oleaginous yeasts under certain culture conditions [1]. Previous studies focused on investigating the lipid production potential and suitable culture conditions of different oleaginous yeast strains by using VFAs as carbon sources. Most of these studies have used low concentrations (2–10 g/L) of acetic acid or VFA mixtures and have prioritized acetic acid as the carbon source [14]. On the one hand, acetic acid is considered superior to other acids for lipid synthesis because of its relatively shorter conversion pathway to produce acetyl-CoA, an important precursor in lipid biosynthesis. On the other hand, high VFA concentrations adversely affect yeast cell growth, sharply reducing the utilization ratio of VFAs and lipid yield. Fei et al. [4] first reported this inhibitory effect in 2011 by using VFA mixtures with an initial concentration higher than 5 g/L as a carbon source to cultivate *C. albidus*. Furthermore, different VFA types and concentrations can produce different intensities of inhibition [15–17]. In general, substrate inhibition on oleaginous microorganisms from high-content VFAs becomes non-negligible when the VFA concentration in culture media exceeds 10 g/L and can even be a fatal factor to cell growth when it exceeds 20 g/L [3, 17]. In previous studies, at appropriate culture conditions, < 2 g/L lipid concentration and 10–40% lipid content were commonly obtained in batch cultivation by using low concentrations (2–10 g/L) of VFAs as a carbon source [17]. To promote the yield of microbial lipids from VFAs, researchers also explored different cultivation modes, such as fed-batch [15, 18], repeated batch [19], sequencing batch [1, 20], continuous cultivation [21], and two-staged cultivation [16, 22]. However, the lipid yields in these studies are unsatisfactory because of the low VFA concentrations. Therefore, substrate inhibition attributed to high VFA concentrations on these oleaginous yeasts has impeded high-lipid-yield production and large-scale applications.

It is commonly accepted that the adverse effects of VFAs on yeast cells are mainly caused by the undissociated form of the acid molecules. When the lipophilic undissociated form of VFAs permeates into the plasma membrane, they dissociate intracellularly into hydrogen ions and the corresponding anions, leading to cytosolic acidification and inducing stress on cell metabolism [23–25]. Yeasts possess a series of regulatory mechanisms to control the entry and exit of hydrogen ions into their cells to maintain a near neutral intracellular environment. However, this capacity is limited for adaptation and regulation [17]. Environmental pH has a crucial effect on the cell proliferation and lipid synthesis of oleaginous microorganisms [26]. Acidic cultivation conditions (pH 5–6) are usually preferred by oleaginous yeasts when glucose is used as a carbon source. In addition, some scholars held the view that alkaline environment might have certain adverse effects on the lipid synthesis of yeasts [4, 6, 27]. Hence, previous researches commonly adopted slightly acidic conditions (pH 5.6–7) rather than alkaline conditions for their cultivation even with VFAs as a carbon source. Considering that the change in carbon source may lead to differences in optimal conditions, the influence of pH has been investigated [4, 28]. However, the best lipid production performance of the strains still occurs under slightly acidic conditions (pH 5.6–7) probably because of the low VFA concentrations and the limited adaptation and regulation capacity of strains. Fei et al. [4] studied the influence of different initial pH of culture media on the biomass and lipid production of *C. albidus* with 2 g/L VFAs as a carbon source and found that the highest lipid concentration and content (0.3 g/L and 26.7%) are achieved at pH 6.0. Gao et al. [17] reported that the cell growth and lipid accumulation of *Yarrowia lipolytica* at an initial pH of 6.0 are highly similar when 2.5 g/L acetic acid, butyric acid, and propionic acid are used as sole carbon sources; this result is also comparable with that of cultures at the same glucose concentration. These results have shown that the adverse effects of VFAs at low concentrations are weak and negligible. However, when high concentrations (> 10 g/L) of VFAs are used as the sole carbon source, their adverse effects should be considered. The acidic pH environment further aggravates this inhibition as VFAs appear largely in an undissociated form under this condition. Liu et al. [3] showed that *C. curvatus* cultured with 30 g/L acetic acid grows much better in an alkaline medium than it does in an acidic medium, but no further systematic research has been conducted. It could be assumed that an alkaline pH environment can effectively alleviate the severe effect of high-content VFA by considerably increasing the degree of dissociation of VFAs in culture media and thus help increase lipid productions. Further work is needed to test this hypothesis.

*Yarrowia lipolytica* is considered a model microorganism for lipid production because of its ability to accumulate high levels of lipids and its suite of efficient genetic tools [29]. Our previous study [17] demonstrated the feasibility of using low VFA concentrations for lipid productions by *Y. lipolytica.* The present study mainly aims to systematically investigate and evaluate the influence of alkaline conditions on alleviating the inhibitory effects of high-content VFAs on oleaginous yeasts for achieving a high cell density and a high lipid yield. First, the adaptable pH range of *Y. lipolytica* when VFAs were used as a carbon source was investigated at a low acetic acid concentration (3 g/L). Then, the VFA utilization, cell growth, and lipid accumulation of *Y. lipolytic* were then evaluated on batch cultures by using high concentrations (30–110 g/L) of acetic acid as the sole carbon source at initial pH of 6, 7, 8, 9, and 10. As two other typical waste-derived VFA types, propionic acid and butyric acid, along with the mixed VFAs (acetic acid: propionic acid: butyric acid = 5:2:3), as carbon sources were also investigated and compared in terms of their feasibilities in cultures. Finally, cultures on undiluted FW and fruit and vegetable waste (FVW) fermentate were prepared to evaluate their potential for practical applications.

## Results and discussion

### Evaluation of the pH tolerance and lipid production of *Y. lipolytica* by using low-concentration acetic acid as a carbon source

pH is an important factor that affects the cell proliferation and lipid synthesis of oleaginous microorganisms [26]. Different microorganisms can adapt to different ranges of environmental pH [17]. The inhibitory effects of weak acids on microorganisms have been ascribed to proton motive force uncoupling and anion accumulation, and the counteracting responses of the yeast cell involve the export of protons and anions at the expense of ATP [30, 31]. Accordingly, the inhibitory effects of VFAs on yeasts are not only concentration dependent but also related to the pH of the environment and to the dissociation degree of VFAs [32].

**Fig. 1:**
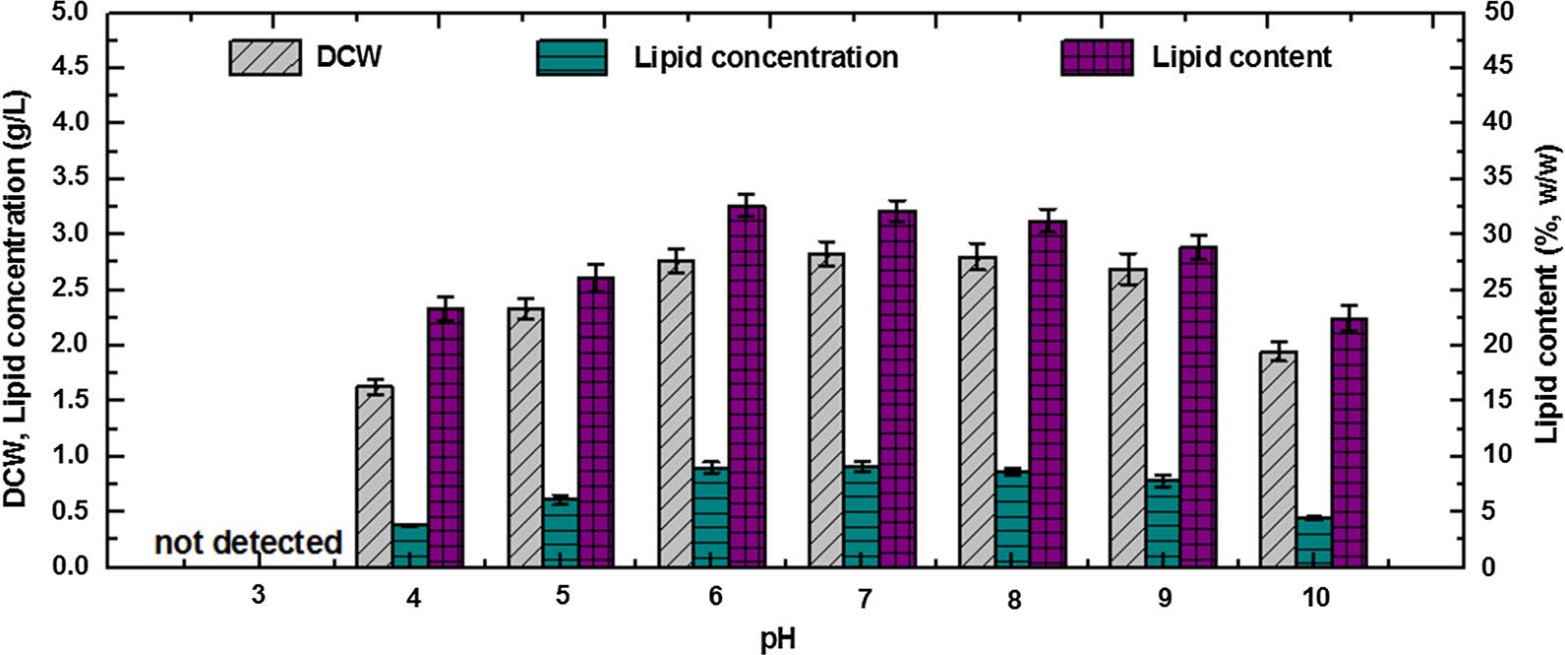
The effect of initial pH on biomass and lipid production by *Y. lipolytica* with 3 g/L of acetic acid as carbon source. *DCW* dry cell weight

Figure 1 illustrates the effects of initial pH on cell growth and lipid accumulation in a batch culture of *Y. lipolytica* by using a low concentration (3 g/L) of acetic acid as the sole carbon source. It is found that from pH 6 to 9, *Y. lipolytica* cells started proliferating without an obvious lag phase (< 3 h) and showed good performance on biomass and lipid production. The highest lipid yield (0.91 g/L) was obtained at pH 7, followed by cultures at pH 6 with a comparable lipid yield of 0.90 g/L. Specifically, the highest biomass production (2.83 g/L) was obtained at pH 7, and the highest lipid content (32.62%) was obtained at pH 6. These results suggested that a slightly acidic pH (6–7) was beneficial to intracellular lipid accumulation, whereas a slightly alkaline pH (7–8) was more advantageous for fast VFA adaptation and cell proliferation. In general, the discrepancies of lipid production from pH 6 to 9 were not obvious. However, for cultures with an initial pH of 5 and 4, biomass and lipid production greatly decreased, and the lag phase was prolonged to approximately 1 and 3 days, respectively. Moreover, no cell growth was observed at pH 3. These phenomena showed that cell growth and lipid accumulation by *Y. lipolytica* was greatly inhibited under such acidic conditions (pH < 5). Given that *Y. lipolytica* could perform well under this acidic condition (pH 4–5) when other carbon sources, such as glucose and glycerol, were used, the inhibitory effect of the unionized form of acetic acid, which prevailed in the acidic environment, should be the most detrimental to growth rather than the unfavorable pH itself. Besides these acidic pH conditions, an initial pH of 10 failed to support good biomass and lipid production, and the lag phase was prolonged to 2–3 days. This result indicated that an excessively alkaline pH (≥ 10) could also be unfavorable to *Y. lipolytica*. VFA consumption was tested after biomass was harvested. Although the specific consumption rate, *Y*_X/S_, and *Y*_L/S_ were different, acetic acid was consumed completely by oleaginous cells in all the the cultures that achieved cell growth within a broad pH range of 4–10. Fei et al. [4] showed that 2 g/L of VFAs is consumed completely by *C. albidus* at neutral pH, whereas only < 50% of VFAs is assimilated at acidic or alkaline pH. The discrepancy may be due to the different strains used for lipid accumulation and reflects the advantage of *Y. lipolytica.*

These results suggested that *Y. lipolytica* could adapt to a wide environment pH (4–10) when low-concentration acetic acid was used as sole carbon source. Cultures with an initial pH from 6 to 9 had good and comparable abilities in cell growth and lipid accumulation. The best lipid production performance occurred at neutral or slightly acidic pH (6–7). These results further indicated that the inhibitory effects of the undissociated acid under a slightly acidic pH (6–7) were quite weak and negligible because of the low concentrations of VFAs and the adaptation and regulation capacity of strains. Thus, neutral and slightly acidic conditions were most commonly adopted in previous studies and recommended by authors when low-concentration (2–10 g/L) VFAs were used as a carbon source.

### Effects of alkaline conditions on the growth and lipid production of *Y. lipolytica* using high-content acetic acid as a carbon source

High concentrations of VFAs cannot be efficiently converted into lipids by oleaginous yeasts [3, 17]. The lethal effect of the high concentrations of VFAs on yeast cells has become a notable bottleneck that prevents high lipid yields and their large-scale applications; hence, further research is needed to solve this problem [14]. Acetic acid is predominantly present in waste-derived VFA mixtures [18] and thus was chosen as a model substrate in this section.

Although the adverse effects of a low VFA concentration could be weak and negligible at pH 6.0 (a classical value used in literature), the toxicity of high VFA concentrations should be considered. Moreover, even a slightly acidic pH environment could further aggravated this inhibition as VFAs predominate in an unionized form in an acidic medium. An alkaline environment could be assumed to effectively alleviate the severe effect of high-content VFAs by significantly increasing the dissociation degree of VFAs in the culture media. The cell growth and lipid accumulation of *Y. lipolytica*, five initial concentrations of acetic acid (30, 50, 70, 90, and 110 g/L), and five levels of initial pH (6, 7, 8, 9, and 10) were tested through batch cultures at 28 °C and 180 rpm to verify this assumption and further study the combined effects of high VFA concentrations and different alkaline conditions on VFA utilization. The results are summarized in Table 1 and analyses are as below.

**Table 1:** Biomass and lipid production of *Y. lipolytica* with high concentrations of acetic acid as carbon source at different pH conditions

Carbon source and initial pH	Biomass (g/L)	Lipid conc. (g/L)	Lipid content (wt%)	*Y*_X/S_ (g/g)	*Y*_L/S_ (g/g)	Lag phase (day)
30 g/L of acetic acid						
pH = 6	10.56 ± 0.73	1.73 ± 0.08	16.41 ± 1.08	0.352 ± 0.024	0.058 ± 0.003	3–5
pH = 7	17.28 ± 0.85	5.16 ± 0.19	29.89 ± 0.89	0.576 ± 0.028	0.172 ± 0.006	0^a^
pH = 8	17.86 ± 0.76	5.24 ± 0.23	29.33 ± 0.86	0.595 ± 0.025	0.175 ± 0.008	0
pH = 9	17.05 ± 0.61	4.57 ± 0.25	26.81 ± 1.07	0.568 ± 0.020	0.152 ± 0.008	0
pH = 10	13.67 ± 0.58	2.75 ± 0.11	20.14 ± 1.46	0.456 ± 0.019	0.092 ± 0.004	0
50 g/L of acetic acid						
pH = 6	N.D.	N.D.	N.D.	N.D.	N.D.	N.D.
pH = 7	27.02 ± 1.21	7.40 ± 0.25	27.38 ± 0.83	0.540 ± 0.024	0.148 ± 0.005	0–1
pH = 8	28.92 ± 1.15	8.01 ± 0.30	27.69 ± 0.96	0.578 ± 0.023	0.160 ± 0.006	0
pH = 9	27.39 ± 1.22	7.10 ± 0.28	25.93 ± 1.35	0.548 ± 0.024	0.142 ± 0.006	0–1
pH = 10	17.07 ± 1.09	3.23 ± 0.09	18.95 ± 0.94	0.341 ± 0.022	0.065 ± 0.002	1–3
70 g/L of acetic acid						
pH = 6	N.D.	N.D.	N.D.	N.D.	N.D.	N.D.
pH = 7	29.10 ± 1.45	6.76 ± 0.36	23.23 ± 1.04	0.497 ± 0.025	0.115 ± 0.006	1–3
pH = 8	37.14 ± 1.56	10.11 ± 0.42	27.22 ± 0.92	0.531 ± 0.022	0.144 ± 0.006	0
pH = 9	35.67 ± 1.64	9.09 ± 0.40	25.49 ± 1.11	0.510 ± 0.023	0.130 ± 0.006	2–3
pH = 10	13.35 ± 0.83	1.84 ± 0.13	13.75 ± 1.50	0.269 ± 0.017	0.037 ± 0.003	3–5
90 g/L of acetic acid						
pH = 6	N.D.	N.D.	N.D.	N.D.	N.D.	N.D.
pH = 7	19.96 ± 0.88	4.36 ± 0.18	21.85 ± 1.01	0.291 ± 0.013	0.064 ± 0.003	1–3
pH = 8	31.02 ± 1.22	7.90 ± 0.37	25.47 ± 1.23	0.417 ± 0.016	0.106 ± 0.005	0–1
pH = 9	24.19 ± 1.19	5.92 ± 0.32	24.46 ± 1.40	0.319 ± 0.016	0.078 ± 0.004	3–4
pH = 10	9.606 ± 0.86	1.38 ± 0.06	14.32 ± 1.07	0.171 ± 0.015	0.024 ± 0.001	6–7
110 g/L of acetic acid						
pH = 6	N.D.	N.D.	N.D.	N.D.	N.D.	N.D.
pH = 7	15.80 ± 0.93	2.49 ± 015	15.75 ± 0.89	0.192 ± 0.011	0.030 ± 0.002	4–5
pH = 8	17.33 ± 0.62	3.85 ± 0.19	22.21 ± 1.28	0.198 ± 0.007	0.044 ± 0.002	2
pH = 9	15.40 ± 0.77	3.30 ± 0.19	21.45 ± 1.53	0.177 ± 0.009	0.038 ± 0.002	3–5
pH = 10	10.20 ± 1.23	1.19 ± 0.10	11.67 ± 1.03	0.167 ± 0.020	0.019 ± 0.002	9–10

All the results presented are the mean values ± SD for three independent replicates

N.D., not detected; *Y*_X/S_, growth yield coefficient, g DCW/g VFAs, *Y*_L/S_, lipid yield coefficient, g lipid/g VFAs

^a^Lag phase less than 3 h was recorded as 0 day

#### Level of acid that *Y. lipolytica* could tolerate and consume

As demonstrated in Table 1, the pH of the cultivation environment had crucial effects on *Y. lipolytica* when high-content acetic acid was used as a carbon source. At an initial pH of 6, only cultures with 30 g/L acetic acid achieved cell growth and lipid accumulation. No cell growth was observed in cultures with other concentrations (50, 70, 90 and 110 g/L) of acetic acid at pH 6, which reflects that the toxicity of such high concentrations of acetic acid under this acidic environment is intolerable to *Y. lipolytica.* However, all the cultures at an initial pH of 7–10 and at 30–110 g/L acetic acid concentrations achieved growth resumption and lipid production. These results demonstrated that increasing the medium pH to alkaline levels greatly alleviated the toxicity of high-content acetic acid and thus greatly increased the acidic level that *Y. lipolytica* could tolerate.

**Fig. 2:**
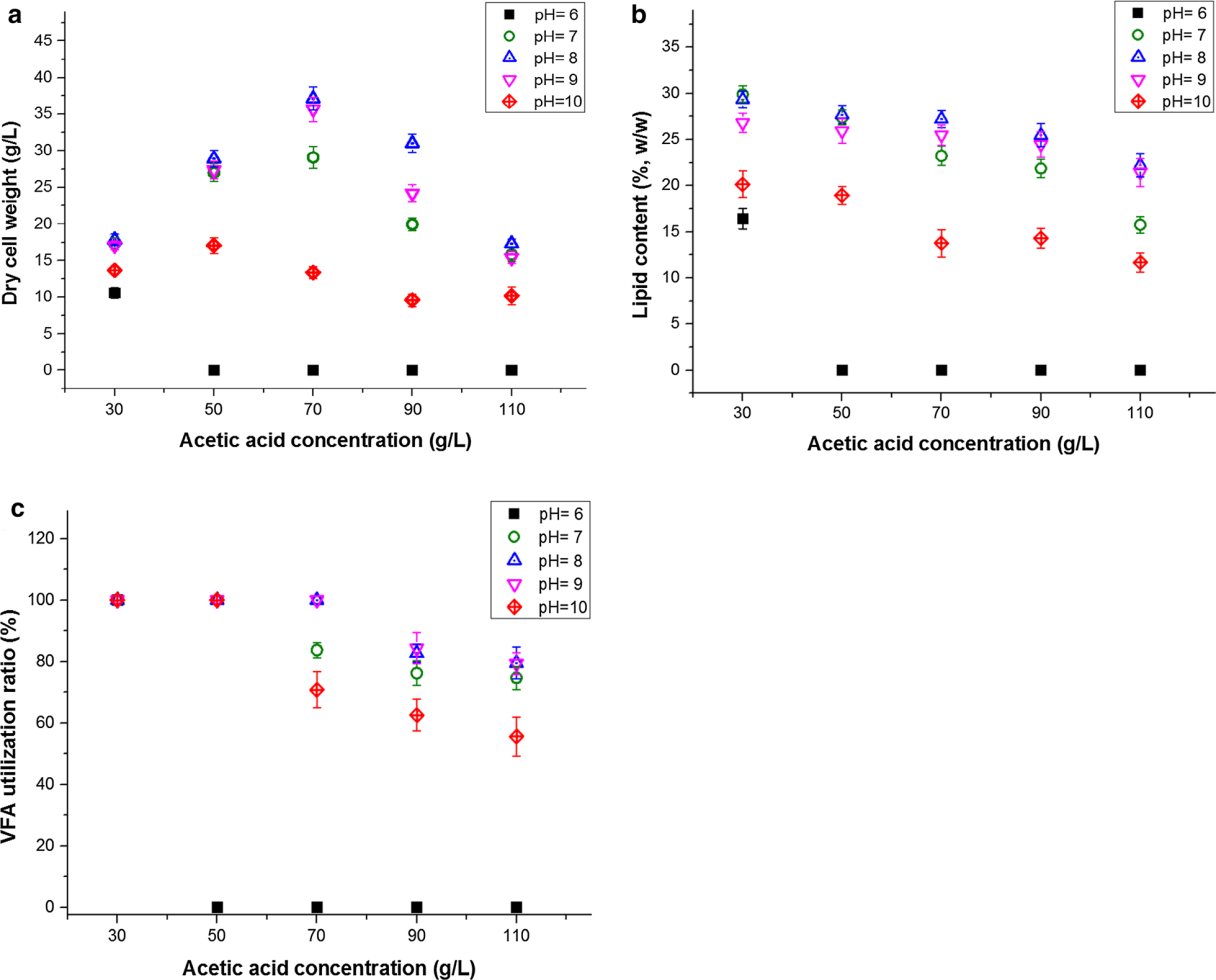
Comparison of **a** dry cell weight, **b** lipid content, and **c** VFA utilization ratio obtained during batch cultivation with different high concentrations of acetic acid as carbon source under different initial pH conditions

Acetic acid consumption by *Y. lipolytica* in all the cultures was tested after the biomass was harvested. The corresponding VFA utilization ratios (%) were calculated (Fig. 2c). When 30 and 50 g/L of acetic acid was used as the sole carbon source, all the cultures that achieved biomass and lipid production consumed acid completely. As the acetic acid concentration increased, the utilization ratios had varying degrees of decline even though acid consumption (g/L) increased accordingly. When 70 g/L of acetic acid was used as a carbon source, only cultures at pH 8 and 9 completely consumed acetic acid, whereas the utilization ratio (%) at pH 7 and 10 declined to 83.73% and 79.84%, respectively. For cultures with 90 and 110 g/L acetic acid, *Y. lipolytica* could not completely assimilate acetic acid at all pH levels, whereas the highest consumption and utilization ratios were obtained at pH 8–9.

It is worth noting that as the acetic acid concentration increased, the optimal medium pH for the growth of *Y. lipolytica* increased to a certain extent. For cultures with 30 and 50 g/L acetic acid, a medium pH of 7–8 was recommended on the basis of biomass and lipid production, whereas cultures at pH 8–9 had improved performances when the concentration of acetic acid was ≥ 70 g/L. However, these results did not indicate that the higher the concentrations of VFA were, the more alkaline the condition would be. From pH 7 to 10, cultures with all these high concentrations of acetic acid as the carbon source had the worst performance at pH 10. This phenomenon was explained by abovementioned conclusions, which elucidated that pH 10 adversely affected the cell growth and lipid accumulation of *Y. lipolytica.*

#### Lag phase and growth curve

Short lag phases were observed in an alkaline environment when high concentrations of acetic acid were used as a carbon source (Table 1). For cultures with 30 g/L acetic acid, approximately 3–5 days of adaptation was needed for the yeast to resume growth at pH 6. However, at pH 7–10, *Y. lipolytica* cells started proliferating without an obvious lag phase (< 3 h), indicating that the adverse effect of acetic acid on cell proliferation was basically eliminated. As the acetic acid concentration increased, the lag phase under different pH conditions was extended to varying degrees. It is found that cultures at pH 8 always had the shortest lag phase. Specifically, at an initial pH of 8, no evident lag phase was observed at ≤ 70 g/L acetic acid. The longest lag phase, which was observed at 110 g/L acetic acid, was achieved only in approximately 2 days. Based on pH 8, the increase and decrease in pH could prolong the period of lag phase. At the same acetic acid concentration, the cultures at pH 10 had the longest lag phase compared with those at pH 7 and 9.

Microorganisms can respond and adapt to harsh environments via a series of adaptive mechanisms. However, this capacity for adaptation and regulation is limited [17]. High acetic acid concentrations can exhibit adverse and even lethal effect on yeast cell growth. Our results showed that an alkaline medium pH could effectively alleviate the growth inhibition and increase the tolerance level of *Y. lipolytica* to high-content acetic acid. Nevertheless, an alkaline environment could not completely eliminate the severe effect of a high acid concentration (> 70 g/L). Moreover, an excessive alkaline environment (pH ≥ 10) negatively affected yeast growth. OD 600 (data not shown) in all the cultures was measured throughout the cultivation duration. Interestingly, the growth curve of *Y. lipolytica* with the imposed non-negligible acid stress under acidic and alkaline conditions exhibited different features. When suffering under severe but tolerable inhibitory effects by acetic acid, *Y. lipolytica* cultured in an alkaline medium could achieve growth resumption after a short-term lag phase, whereas the slope of the growth curve in the exponential phase was usually not large. On the contrary, *Y. lipolytica* cultured in an acidic medium needed a much longer period of adaptation to resist growth inhibition if the inhibitory effect was tolerable. However, when growth was resumed, the slope of the growth curve in the exponential phase was larger than that in the cultures in an alkaline medium. These discrepancies could be explained by the different inhibitory mechanisms of acid and yeast responses under different pH conditions. The inhibitory effects of VFAs under alkaline conditions, which mainly stem from anion (dissociated weak acid) accumulation or excessive alkaline pH, were usually not lethal. The strain could easily resist damage, but the cell proliferation rate was likely impaired because of low vitality. However, in the case of cultures under acidic conditions, the strain experienced difficulty in surviving because of inhibitory effects due to cytosolic acidification by uncoupling could be fatal. Under this extreme circumstance, the adaptation period is markedly prolonged and usually accompanied by changes in membrane structure and gene mutations [30–33]. Once adaptation was successful, the growth resumption would proceed at a fast cell proliferation rate.

#### Biomass and lipid production

The effects of different initial pH values on the biomass production of cultures with high-content acetic acid as a carbon source are illustrated in Fig. 2a. In accordance with the results in “Level of acid that *Y. lipolytica* could tolerate and consume” and “Lag phase and growth curve” sections, *Y. lipolytica* could accumulate more biomass under alkaline conditions of pH 7–9 and obtain the highest biomass at pH 8. Acetic acid concentration considerably affected biomass production. With concentrations < 70 g/L, the increase in acetic acid concentration caused an increase in the biomass, whereas cultures with > 70 g/L acetic acid had a decreased biomass. The highest biomass production (37.14 g/L) was obtained with 70 g/L acetic acid at an initial pH of 8.

These results indicated that the alkaline condition for alleviating the inhibitory effects of high-concentration VFAs had a certain concentration limit. This limitation was related to the mechanism of the remission effect of an alkaline environment. This mechanism involved alleviating the inhibitory effect attributed to uncoupling by significantly increasing the dissociation degree of weak acids. However, this mechanism could not work on anion accumulation. It is generally accepted that the lethal effects of weak acids occur due to the lowering of microorganism’s internal pH by uncoupling, while some researchers argue that the intracellular accumulation of the dissociated weak acid (anion) itself plays the most significant role in cell death [32]. The results in the present study indicated that uncoupling plays a major role in the lethal effect on *Y. lipolytica* cultured with VFAs, whereas anion accumulation could have an adverse effect only when its concentration was high.

As shown in Fig. 2b, for cultures at all the pH conditions, the highest lipid content was obtained with 30 g/L, from pH 6 to 10, which was 16.41, 29.89, 29.33, 26.81 and 20.14%, respectively. In contrast to the changes observed in biomass, the lipid content showed a trend of unilateral decline as the acetic acid concentration increased. This trend was consistent with our previous study [17], which involved culturing *Y. lipolytica* with low concentrations (< 20 g/L) of VFA under a slightly acidic pH of 6. However, under alkaline conditions in this study, the downward trend of the lipid content was much gentle. In particular, at pH 8 and 9, the cultures with 110 g/L acetic acid could maintain > 20% of their lipid content. On the contrary, under slightly acidic and neutral conditions of pH 6 and 7, *Y. lipolytica* lipid accumulation was clearly inhibited as the acid concentration increased.

In general, lipid production depends on both biomass and lipid content. When the acid concentration increased from 30 to 110 g/L, the obtained lipid concentration initially increased and then decreased (Table 1). The highest lipid production (10.11 g/L) was obtained with 70 g/L acetic acid at an initial pH of 8. Notably, besides the increased biomass and lipid production, the growth yield coefficient (*Y*_X/S_) and lipid yield coefficient (*Y*_L/S_) of *Y. lipolytica* cultured with such high acetic acid concentrations were greatly improved under the proper alkaline conditions determined in this study. With ≤ 70 g/L acetic acid, *Y*_X/S_ could exceed 0.50, and *Y*_L/S_ could exceed 0.11 by adjusting the medium pH to 7–9. However, in our previous study [17], at pH 6, cultures with 20 g/L acetic acid could only achieve *Y*_X/S_ of 0.37 and *Y*_L/S_ of 0.05. The significant improvement of *Y*_X/S_ and *Y*_L/S_ further elucidated that the increased biomass and lipid yield was not only because of the abundant carbon source, but more importantly, due to the improved viability and vitality of *Y. lipolytica* under alkaline conditions against the imposed acid stress.

#### Change in pH during cultivation

**Fig. 3:**
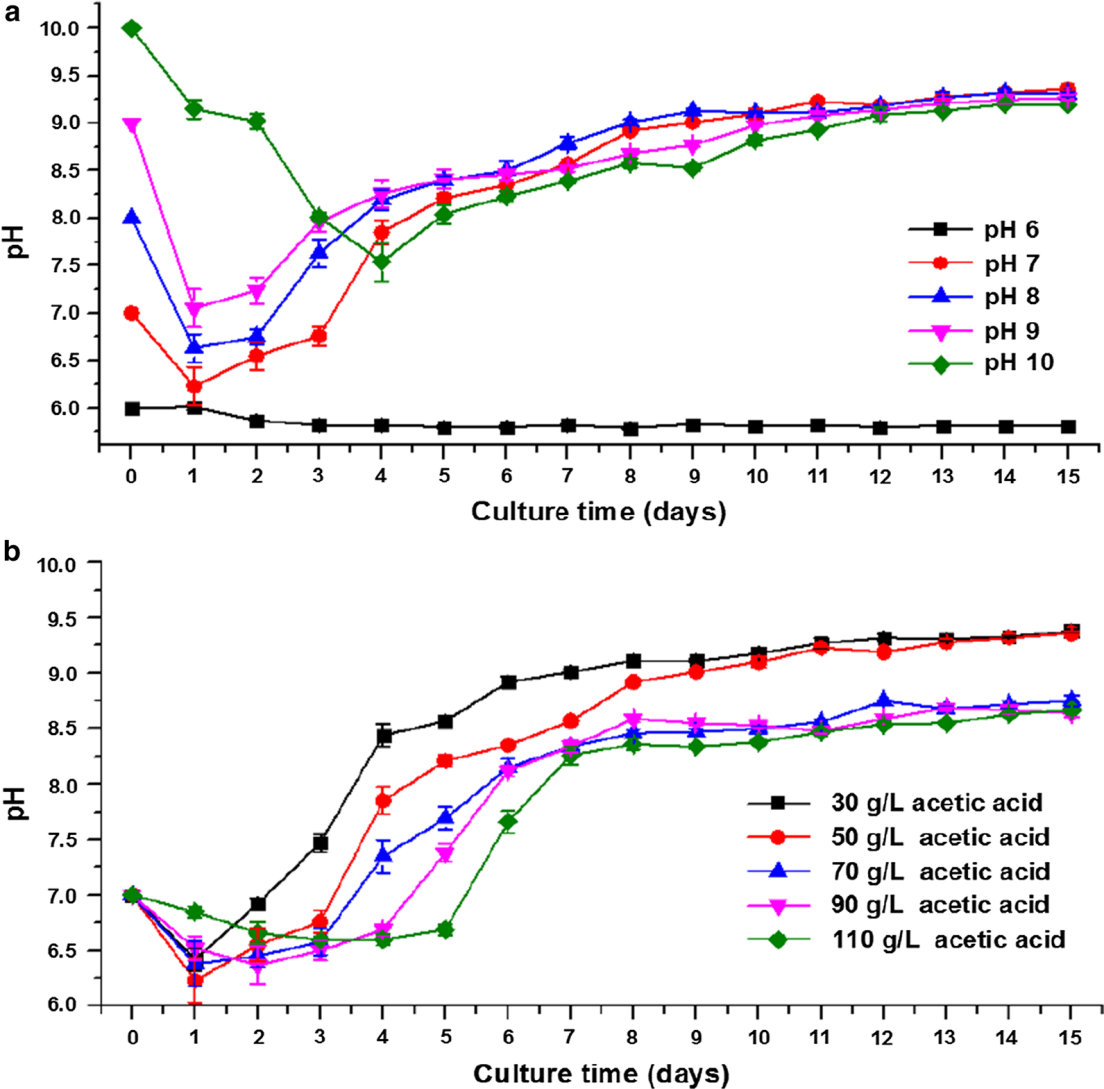
The change of pH in cultures **a** with 50 g/L of acetic acid at different initial pH, **b** with 30–110 g/L of acetic acid at an initial pH of 7

The nutrient consumption and metabolism of yeast can change the pH in culture media. The pH of all the cultures was determined every day after inoculation. It is found that the pH of the cultures that did not have cell growth remained almost unchanged during the cultivation period. The cultures showing biomass and lipid production demonstrated similar trends in pH change. Figure 3a shows the changes in the pH of the cultures with 50 g/L acetic acid at different initial pH values. As depicted, the pH of the cultures at an initial pH of 6 remained almost unchanged. The pH in all of the cultures at an initial pH of 7–10 had varying degrees of decline to reach the lowest point on the first few days, and then gradually increased, and finally stabilized at pH 9.1–9.4. Lian et al. [6] and Zheng et al. [28] indicated that the massive uptake of ammonium leads to a rapid pH decrease on the first few days, whereas the consumption of acetate ions (CH_3_COO^−^) and the production of OH^−^ groups result in a pH increase on the following days.

In the current study, the lowest pH of the cultures varied with different initial pH values. Notably, for cultures at an initial pH of 7, the lowest pH point could nearly drop to 6 (pH 6.23 ± 0.20). Acetic acid, propionic acid, and butyric acid have pKa of 4.76, 4.87, and 4.82, respectively, indicating that 99% of these VFAs appear in the dissociated form at pH 7. However, such a decrease in pH to an acidic level at the early stage of cultures could largely increase the amount of undissociated VFAs in the system and their inhibitory effects. This inhibition could adversely affect yeast cell proliferation and lipid accumulation, causing the system pH to change slowly, prolonging the lag phase, and changing lipid production performance. Figure 3b illustrates the changes in the pH of the cultures with 30–110 g/L acetic acid at an initial pH of 7. This inhibition and its adverse effect were more obvious in cultures with a higher acetic acid concentration. This occurrence could also explain that the optimal initial pH was more alkaline to a certain extent to avoid the inhibitory effect from the undissociated acid caused by a drop in medium pH when the acetic acid concentration was too high (> 70 g/L).

As shown in Fig. 3a, irrespective of the initial pH or changes in pH during the cultivation period, the final fermentation pH of cultures which realize biomass and lipid accumulation reached at an equivalent level. This result was inconsistent with studies by Santamauro et al. [34] and Zheng et al. [28], who obtained different final pH values by using other types of organic waste to cultivate *M. pulcherrima* and *C. sorokiniana* at different initial pH values, respectively. This discrepancy may be due to the different strains and feedstock used, and further metabolic level studies are needed to elucidate the specific mechanisms of *Y. lipolytica* on regulating intracellular and extracellular pH when VFAs are used as carbon sources. Lian et al. [6] revealed that the initial acetate concentration unlikely affects the final fermentation pH when fermentation is carried out with *C. curvatus*. In the present work, as demonstrated in Fig. 3b, the initial VFA concentration did not affect the final pH in the cultures that completely consumed VFAs (cultures with 30 and 50 g/L acetic acid). The cultures with incomplete VFA consumption resulted in a relatively low final fermentation pH.

### Growth and lipid production of *Y. lipolytica* using high-content propionic acid, butyric acid, and mixed VFAs as carbon sources under an alkaline condition

**Fig. 4:**
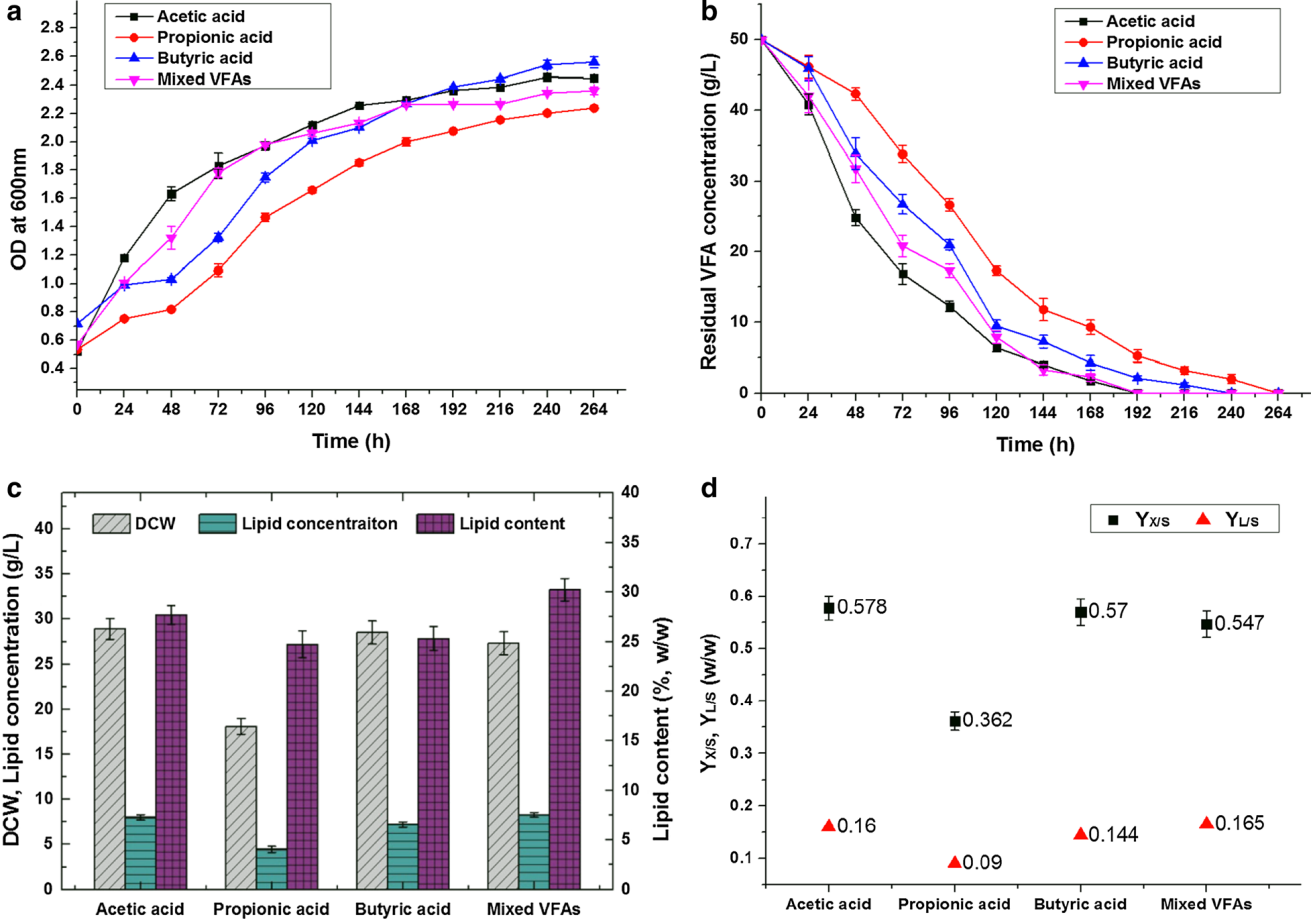
Profiles of **a** growth curve, **b** VFA consumption, **c** biomass and lipid production, and **d** growth yield coefficient (*Y*_X/S_) and lipid yield coefficient (*Y*_L/S_) obtained during batch cultivation with 50 g/L of different types of VFA as carbon source at an initial pH of 8.0

**Fig. 5:**
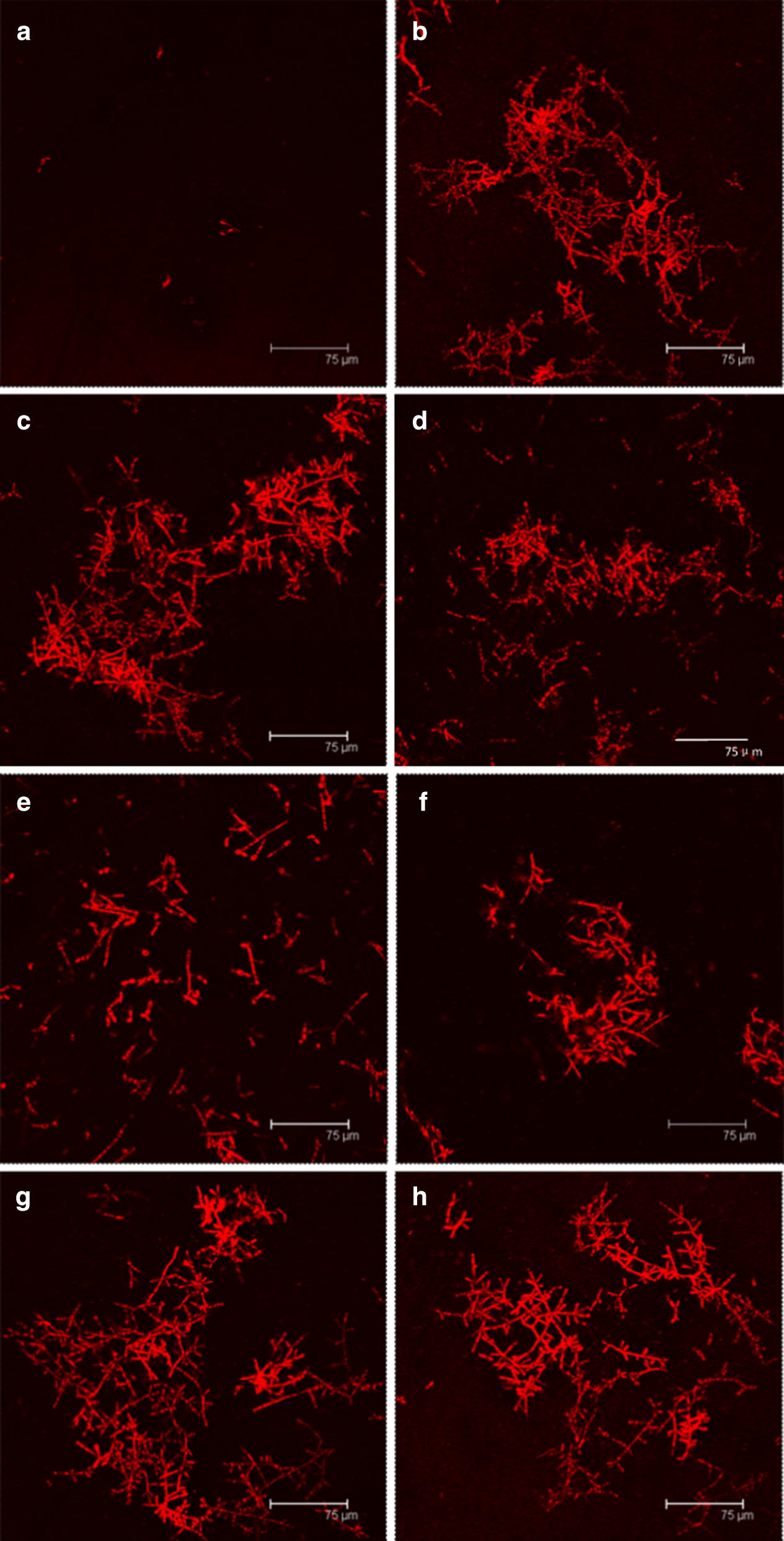
Confocal fluorescent images of *Y. lipolytica* cultured with 50 g/L of acetic acid at an initial pH of **a** 6.0, **b** 7.0, **c** 8.0, **d** 9.0, **e** 10.0, and with 50 g/L of **f** propionic acid, **g** butyric acid, **h** mixed acids (acetic: propionic: butyric acid = 5:2:3) at an initial pH of 8.0. The scale bar is 75 μm

The concentration of waste-derived VFAs is generally no more than 50 g/L, and the composition ratio of VFAs can be affected by substrate types and fermentation conditions, with acetic acid, propionic acid, and butyric acid as the main components [8–13]. Batch cultures were conducted by using 50 g/L propionic acid, butyric acid, and a synthetic acetic acid/propionic acid/butyric acid (5:2:3) VFA mixture as carbon sources with an initial pH of 8 to evaluate the feasibilities of the two other typical VFA types and a mixed system. The results are illustrated in Figs. 4 and 5.

The growth curve (Fig. 4a) showed that all of the batches started cell proliferation without an evident lag phase, indicating that the growth inhibitory effects of these high-content VFAs on *Y. lipolytica* were effectively relieved under this alkaline condition. The cell growth rates on acetic acid and mixed acid were slightly greater than those on propionic acid and butyric acid during the exponential phase. The differences in cell growth rates were directly associated with the different utilization rates of VFAs (Fig. 4b), which were attributed to various metabolic pathways of different single VFA after intake. Acetic acid can be directly transformed to acetyl-CoA, a central intermediate in lipid synthesis [20]. Propionate, an odd-chain carboxylic acid, should be converted to propionyl-CoA and then enter the tricarboxylic acid cycle via the interconversion of methylmalonyl-CoA to succinyl-CoA [7]. Butyrate must undergo a number of biochemical transformations, including β-oxidation to acetoacetyl-CoA, and further cleave to acetyl-CoA [35]. In our study, the biomass and lipids produced were harvested at the end of the stationary phase. As demonstrated in Fig. 4c, the cultures on acetic acid, butyric acid, and mixed acids had comparable biomass and lipid production, whereas the cultures on propionic acid showed a much inferior performance, especially biomass production. The concentrations of the biomass produced in the cultures with acetic acid, butyric acid, and mixed acid were 28.92, 28.52, 27.33 g/L, respectively. By comparison, the concentration of the biomass produced in the cultures with propionic acid was only 18.11 g/L. The lipid contents slightly differed. The highest lipid content was observed in mixed acid (30.25%), followed by acetic acid (27.69%), butyric acid (25.32%), and propionic acid (24.73%). Thus, the cultures with 50 g/L acetic acid, propionic acid, butyric acid, and mixed acids at an initial pH of 8 achieved 8.01, 4, 48, 7.22, and 8.27 g/L lipid yields, respectively.

*Yarrowia lipolytica* prefers acetic acid to propionic acid and butyric acid for cell growth and lipid accumulation [17]. In our previous research, 2.5–20 g/L VFAs were used as carbon sources in a slightly acidic environment (pH = 6), and our results revealed that the inhibitory effects of the same concentrations of propionic acid and butyric acid were much more severe than those of acetic acid. Thus, *Y. lipolytica* could only tolerate low concentrations of propionic acid (≤ 10 g/L) and butyric acid (≤ 5 g/L) with low biomass and lipid production. However, an alkaline medium (pH = 8) was used in this study, so the levels of propionic acid and butyric acid that *Y. lipolytica* could tolerate and the corresponding biomass and lipid production remarkably improved. Interestingly, *Y*_X/S_ (0.570) and *Y*_L/S_ (0.144) of *Y. lipolytica* using butyric acid as its carbon source reached a comparable level with those of *Y. lipolytica* utilizing acetic acid as its carbon source (0.578, 0.160). Thus, comparable biomass and lipid yields were obtained. According to our supplementary experimental results at other pH levels (pH 6–10, Table 2), the performance of the cultures at pH 8–10 was better than that of the cultures at pH 7 when 50 g/L butyric acid was used as a carbon source. Conversely, the performance of the cultures at pH 7–9 was better than that of the cultures at pH 10 when acetic acid and propionic acid were used as carbon sources. Notably, the cultures with butyric acid at pH 10 could still perform well. These results indicated that a more alkaline pH compared with that of acetic acid and propionic acid was beneficial to the bioconversion of high-content butyric acid to lipids by *Y. lipolytica*.

**Table 2:** Biomass and lipid production of *Y. lipolytica* with 50 g/L of propionic acid, butyric acid and mixed VFAs as carbon source at different pH conditions

Carbon source and initial pH	Biomass (g/L)	Lipid conc. (g/L)	Lipid content (wt%)	*Y*_X/S_ (g/g)	*Y*_L/S_ (g/g)	Lag phase (day)
50 g/L of propionic acid						
pH = 6	N.D.	N.D.	N.D.	N.D.	N.D.	N.D.
pH = 7	15.99 ± 0.92	3.83 ± 0.21	23.96 ± 1.34	0.320 ± 0.018	0.077 ± 0.004	1–2
pH = 8	18.11 ± 0.87	4.48 ± 0.34	24.73 ± 1.20	0.362 ± 0.017	0.090 ± 0.007	0^a^
pH = 9	17.26 ± 1.01	4.33 ± 0.35	25.06 ± 1.47	0.345 ± 0.020	0.087 ± 0.007	0–1
pH = 10	9.67 ± 0.69	1.58 ± 0.16	16.33 ± 1.33	0.193 ± 0.014	0.032 ± 0.003	2–3
50 g/L of butyric acid						
pH = 6	N.D.	N.D.	N.D.	N.D.	N.D.	N.D.
pH = 7	25.45 ± 1.10	6.53 ± 0.24	25.65 ± 1.17	0.509 ± 0.022	0.131 ± 0.005	1–3
pH = 8	28.52 ± 1.28	7.22 ± 0.27	25.32 ± 1.20	0.570 ± 0.026	0.144 ± 0.005	0
pH = 9	28.92 ± 1.28	7.92 ± 0.23	27.39 ± 1.47	0.578 ± 0.026	0.158 ± 0.005	0
pH = 10	26.86 ± 1.43	7.16 ± 0.30	26.67 ± 1.33	0.537 ± 0.029	0.143 ± 0.006	1–2
50 g/L of mixed VFAs (acetic: propionic: butyric acid = 5:2:3)						
pH = 6	16.93 ± 0.60	3.16 ± 0.17	18.66 ± 1.30	0.339 ± 0.012	0.063 ± 0.003	2–3
pH = 7	27.48 ± 1.18	8.32 ± 0.33	30.28 ± 1.34	0.550 ± 0.024	0.166 ± 0.007	0
pH = 8	27.33 ± 1.26	8.27 ± 0.26	30.25 ± 1.13	0.547 ± 0.025	0.165 ± 0.005	0
pH = 9	26.94 ± 1.30	8.28 ± 0.44	30.72 ± 1.11	0.539 ± 0.026	0.166 ± 0.009	0
pH = 10	23.65 ± 1.29	5.33 ± 0.22	22.53 ± 1.42	0.473 ± 0.026	0.107 ± 0.004	1–3

All the results presented are the mean values ± SD for three independent replicates

N.D., not detected; *Y*_X/S_, growth yield coefficient, g DCW/g VFAs, *Y*_L/S_, lipid yield coefficient, g lipid/g VFAs

^a^Lag phase less than 3 h was recorded as 0 day

A mixture of acetic acid, propionic acid, and butyric acid (5:2:3) was used as sole carbon sources with a total VFA concentration of 50 g/L to evaluate acid utilization and lipid production by *Y. lipolytica* using high-content waste-derived VFAs. The VFA ratio was identified on the basis of our previously reported results focusing on the anaerobic fermentation of FW materials [17]. Although certain levels of unfavorable acids, such as propionic acid, were found, the cultures with mixed VFAs favored good growth and lipid accumulation profiles. In Fig. 4c, the cultures with the mixed VFAs had the highest lipid yield (8.27 g/L) and the highest lipid content (30.25%). The biomass production on mixed VFAs was 27.33 g/L. This value was slightly lower but still comparable with that in cultures with single acetic acid and butyric acid, which were 28.92 and 28.52 g/L, respectively. In addition, our supplementary experimental results (Table 2) indicated that the cultures with mixed VFAs could still resume growth after 2–3 days of adaptation and finally obtain a lipid yield of 3.16 g/L even at an initial pH of 6. No cell growth and lipid accumulation were observed in the cultures with a single acid under the same condition. These phenomena involved the diversity of carbon source and the specific VFA utilization of *Y. lipolytica* in a mixed VFA system. The utilization of different single VFAs by yeasts would be interactively influenced in a mixed VFA system. Some researchers reported that different types of acids could be used simultaneously [19], while some also found yeasts would preferentially utilize acetic acid followed by propionic and butyric acid [36]. It was found in our previous study [17] that the utilization of different acids by *Y. lipolytica* in a mixed VFA system occurs in a step-wise manner, not in a synchronized manner. *Y. lipolytica* first uses acetic acid for lipid production. The metabolism of propionate and butyrate by *Y. lipolytica* is likely suppressed or halted when sufficient acetate is available as a carbon source. Therefore, acetic acid in VFA mixtures, which did not reach lethal concentration, could be metabolized first to obtain a certain level of cell density, thus further promoting the follow-up metabolism of residual VFAs. Moreover, different acids as carbon sources can induce general and acid-specific responses of yeast and increase phenotypic cell-to-cell heterogeneity [30]. Consequently, the likelihood that cells asynchronously resume growth during the acid-adaptation phase is increased, possibly contributing to a stronger acid tolerance of *Y. lipolytica* and a shorter lag phase than that of cultures on a single type of VFA. Meanwhile, the slightly lower biomass production on mixed VFAs than single acid could also be explained by the increase in phenotypic cell-to-cell heterogeneity, as it could be a result of the accumulation of cells that persisted in a viable but non-proliferating state.

### Biomass and lipid production of *Y. lipolytica* on FW and FVW fermentate that contained VFAs under alkaline conditions

With a high soluble organic matter content, FW and FVW are easily degraded and can rapidly produce large amounts of VFAs during hydrolysis and acidification. Table 3 shows the characteristics of FW and FVW fermentate used by *Y. lipolytica* as feedstock for lipid production. As demonstrated in Table 4, the cultures with FW and FVW fermentates with an initial pH of 6.0 had a 48–72 h lag phase prior to an effective cell growth and resulted in an inferior performance on biomass and lipid production. Moreover, the VFAs in FW fermentate could not be fully consumed. These results indicated that the viability and vitality of yeasts were inhibited in this initial acidic environment. By increasing the initial pH to 7 and 8, the yeasts could start proliferating with almost no lag phase (< 3 h) and completely utilize VFAs. The biomass and lipid production were also significantly improved. The maximum lipid production of both cultures on FW and FVW fermentates were obtained at an initial pH of 8, indicating that this initial alkaline environment was also beneficial to cultures with organic waste-derived VFAs. The highest biomass and lipid production in the cultures with FW fermentate reached 14.65 g/L (*Y*_X/S_: 0.414) and 3.20 g/L (*Y*_L/S_: 0.091) with a lipid content of 21.86%, respectively. Conversely, the highest biomass and lipid production in the cultures with FVW fermentate were 11.84 g/L (*Y*_X/S_: 0.534) 3.08 g/L (*Y*_L/S_: 0.139) with a lipid content of 26.02%, respectively.

**Table 3:** Characteristics of the supernatant of FW and FVW fermentate using as the feedstock for lipid production by *Y. lipolytica*

Item	Concentration (g/L)	
	FW	FVW
SCOD	56.76 ± 2.22	25.93 ± 1.59
Total nitrogen	2.14 ± 0.08	0.32 ± 0.03
Total VFA	35.35 ± 1.45	22.18 ± 1.07
Acetic acid	16.11 ± 1.02	5.34 ± 0.87
Propionic acid	6.69 ± 0.55	1.02 ± 0.30
Butyric acid	10.75 ± 0.20	14.23 ± 0.42
Valeric acid	0.83 ± 0.06	0.92 ± 0.05
Isobutyric acid	0.35 ± 0.03	0.21 ± 0.02
Isovaleric acid	0.62 ± 0.02	0.46 ± 0.02

The concentration of the carbon and nitrogen sources in the effluent were measured after the solid content was removed by centrifugation and filtration. Three samples from the same experiment were analyzed

**Table 4:** Biomass and lipid production of *Y. lipolytica* on FW and FVW fermentate at different pH conditions

Carbon source	VFA from FW fermentate			VFA from FVW fermentate		
Initial pH	6.0	7.0	8.0	6.0	7.0	8.0
VFA utilization ratio	79.80%	100%	100%	100%	100%	100%
DCW (g/L)	9.12 ± 0.59	14.23 ± 0.77	14.65 ± 0.55	6.77 ± 0.42	10.52 ± 0.50	11.84 ± 0.63
Lipid conc. (g/L)	1.35 ± 0.14	3.06 ± 0.40	3.20 ± 0.37	1.19 ± 0.11	2.77 ± 0.28	3.08 ± 0.28
Lipid content (wt%)	14.78 ± 1.73	21.52 ± 1.51	21.86 ± 1.45	17.58 ± 1.16	26.33 ± 0.92	26.02 ± 1.04
*Y*_X/S_	0.323 ± 0.020	0.403 ± 0.021	0.414 ± 0.015	0.305 ± 0.018	0.474 ± 0.023	0.534 ± 0.028
*Y*_L/S_	0.048 ± 0.005	0.087 ± 0.011	0.091 ± 0.010	0.054 ± 0.005	0.125 ± 0.012	0.139 ± 0.012
Lag phase (d)	2–3	0	0	3	0	0

^a^Lag phase less than 3 h was recorded as 0 day

In contrast to FVW fermentate, FW fermentate with an initial pH of 8 did not show evident advantages over cultures with an initial pH of 7. This result was probably due to the much higher nitrogen concentration of FW fermentate (TN: 2.14 g/L) than that of FVW fermentate (TN: 0.32 g/L). With an enhanced alkaline environment, ammonia–nitrogen tended to exist in the form of free ammonia, which could permeate into cells and induce stress on cell metabolism at high concentrations. The comparison of the cultures with FW and FVW fermentates revealed that the lag phase duration of the cultures with FW fermentate at pH 6.0 was shorter than that of the cultures with FVW fermentate, although the VFA concentration of FW fermentate (total VFAs: 35.35 g/L) was much higher than that of FVW fermentate (total VFAs: 22.18 g/L). This result could also be ascribed to the higher nitrogen concentration in the FW fermentate than in the FVW fermentate. In general, sufficient nitrogen is beneficial to yeast cell proliferation and growth. However, microbial lipids always accumulate under nitrogen-starved conditions [37]. C/N has a critical effect on lipogenesis [38]. Oleaginous yeasts have difficulty in yielding a high lipid content at an initial C/N ratio lower than 20/1, and C/N ratios from 40/1 to 80/1 generally seem to be appropriate for lipid accumulation in most oleaginous microorganisms [39]. With an initial C/N of 7.76, the cultures with FW fermentate had much lower lipid contents and *Y*_L/S_ than those with FVW fermentate with an initial C/N of 35.38. In addition, the presence of unknown inhibitors in FW fermentate due to its complex composition may also adversely affect yeast lipogenesis [17].

### Fatty acid compositional analysis of lipids

The fatty acid composition of lipids obtained during the batch cultivation of *Y. lipolytica* with high acetic acid concentrations induced by an alkaline pH of 8 and those with low acetic acid concentrations at pH of 6 was determined through GC analysis after esterification. The results are illustrated in Table 5. On the one hand, the accumulated fatty acids of the lipids in both cases were predominant with carbon chain lengths of C16 and C18, corresponding to those of vegetable and soybean oil [40]. On the other hand, the fatty acids of the lipids obtained in both cases are mostly unsaturated, with oleic acid (C18:1) acting as the major component, which is highly suitable for biodiesel production [17]. Although the ratio of C16–C18 of fatty acids obtained under alkaline conditions with high-content acetic acid decreased to 80.62% compared with that in the cultures with low-content acetic acid at pH 6, the fatty acid composition had a wider variety. In particular, the content of odd-numbered fatty acids (C15, C17) slightly increased. The produced odd-numbered fatty acids can be applied to cosmetics, pesticide formulation, health care, and other aspects and thus have increased potential for various applications [41].

**Table 5:** Main fatty acid composition of the lipids produced by *Y. lipolytica* under different culture conditions

Relative fatty acid content (%)	Carbon source and initial pH	
	50 g/L acetic acid at pH 8.0	3 g/L acetic acid at pH 6.0
Total C16 and C18	80.62	89.26
Unsaturated C16-C18	60.61	71.87
Palmitic acid (C16:0)	10.37	13.18
Palmitoleic acid (C16:1)	14.81	8.55
Stearic acid (C18:0)	9.64	4.21
Oleic acid (C18:1)	37.11	40.82
Linoleic acid (C18:2)	8.36	22.5
Lionlenic acid (C18:3)	0.33	N.D.
Total C15 and C17	6.05	0.95
Ginkgolic acid (C15:0)	0.76	N.D.
Ginkgolic acid (C15:1)	3.31	N.D.
Ginkgolic acid (C17:0)	0.21	N.D.
Ginkgolic acid (C17:1)	1.77	N.D.

Some other fatty acids (C14, C20, C22) were also detected in trace amount and were not included in this table

## Conclusions

In contrast to cultures with low concentrations (< 10 g/L) of VFA, when high concentrations of VFAs as the carbon source were used, the adverse effects of VFAs ascribed to uncoupling became the dominating inhibitory factor. Thus, the slightly acidic conditions (pH 5.6–7), which were considered the most favorable to oleaginous yeast cultivation, were inappropriate for cultures with a high-content VFA because it could markedly aggravate inhibition caused by the unionized form of VFAs.

This study assumed and verified that alkaline conditions (pH 7–9) could effectively alleviate the lethal effect of high-content VFAs on *Y. lipolytic* to achieve a high cell-density and high lipid yield. An initial pH of 8 was determined as the optimal pH condition for the bioconversion of high-content VFAs to lipids by *Y. lipolytica*. Under this alkaline condition, cultures with high-content butyric acid could have comparable biomass and lipid production with cultures with acetic acid, whereas the performance with propionic acid was much inferior to that of the other acids. Mixed VFAs were more beneficial to fast adaptation and lipid production than single types of VFAs. Furthermore, feasibilities on undiluted FW and FVW fermentate were evaluated, and meaningful information was provided for practical application. In summary, this study could provide an effective strategy and useful information for the efficient bioconversion of high-content waste-derived VFAs into microbial lipids.

## Materials and methods

### Strain and inoculum preparation

*Yarrowia lipolytica* (CICC 31596) was obtained from the China Center of Industrial Culture Collection. For strain preservation, the yeast was maintained at 4 °C on YPD agar slants (2 mass% agar powder) and subcultured monthly. The YPD medium contained 20 g/L glucose, 20 g/L peptone, and 10 g/L yeast extract.

For the pre-culture of the seed cells, a loopfull of yeast cells were inoculated in 100 mL of YPD medium in a 250 mL flask and then incubated in a rotary shaker set to 180 rpm at 28 °C for 24 h. The resulting cultures were re-inoculated in the YPD medium at a ratio of 10% (v/v) and cultivated under the same conditions for 24 h as the seed culture.

### Culture conditions

#### Cultures on synthetic VFAs

Batch culture experiments were performed in 100 mL of fermentation medium in 250 mL Erlenmeyer flasks. The nutrient medium used for all the cultures contained (g/L): KH_2_PO_4_, 3; NH_4_Cl, 1; MgSO_4_7H_2_O, 1; FeCl_3_·6H_2_O, 0.015; ZnSO_4_·7H_2_O, 0.0075; and CuSO_4_·5H_2_O, 0.0005. In addition to the nutrient medium, acetic acid, butyric acid, propionic acid, and a mixture of these VFAs were used as sole carbon sources. The seed culture medium was inoculated in the fermentation medium at a ratio of 10% (v/v). The pH of the fermentation medium was adjusted with 2 mol/L NaOH and HCl solutions when needed.

#### Cultures on FW and FVW fermentate

The anaerobic fermentation of FW and FVW was performed in an anaerobic digestion reactor at a working volume of 1.5 L. FW was derived from the Dongcun solid waste treatment plant, which collects food waste from restaurants in Beijing, to prepare the feed. FVWs were collected from a market in the University of Science and Technology Beijing. Material processing and anaerobic fermentation were performed in accordance with a previous method [17].

After 5 days of fermentation, when hydrolysis and acidogenesis were finished, the effluent was collected and centrifuged at 5000 rpm for 10 min to remove the suspended solids. The liquid supernatant was filtered through a 0.45 μm membrane. The supernatant of FW and FVW fermentation effluent was then used as the feedstock without dilution or any additional nutrients for lipid production by *Y. lipolytica*. The culture was conducted in 250 mL Erlenmeyer flasks that contained 100 mL of medium at an initial pH of 6.0, 7.0, and 8.0.

The equipment and medium used in this study were steam autoclaved at 121 °C for 20 min before inoculation. All batch cultures were incubated in a rotary shaker at 180 rpm and 28 °C. This procedure was performed in triplicate.

### Analytical methods

#### Analysis of FW and FVW fermentate

For FW and FVW fermentation, TS, VS, and pH were measured in accordance with the standard method [42]. Suspended solids were separated from the fermentation effluent via centrifugation at 5000 rpm for 10 min. The liquid supernatant was filtered through a 0.45 μm membrane. Soluble COD (SCOD), total nitrogen (TN), and VFAs were then measured. SCOD and TN were analyzed via the HACH method. VFAs were determined as described in “VFA utilization” section.

#### Cell growth and biomass production

During cultivation, cell concentration was determined as the absorbance of the culture broth at 600 nm (OD_600_) to describe the cell growth curve. At the end of the stationary phase, biomass was harvested for lipid extraction and the corresponding measurements.

Dry cell weight (DCW) was determined to describe biomass production. A 10 mL culture broth sample was centrifuged at 8000 rpm for 10 min. The cell pellet was washed twice with distilled water, dried to constant weight in an oven at 105 °C, and weighed [43].

#### VFA utilization

After the culture broth was centrifuged, the residual VFAs in the liquid supernatant were determined to describe the utilization of VFAs by the yeast. First, the liquid supernatant was filtered through a 0.45 μm membrane. The concentrations of VFAs were then analyzed with a gas chromatograph (Shimadzu, GC-2014) fitted with a capillary column (Stabilwax-DA, 30 m × 0.25 mm × 0.25 μm) and a flame ionization detector. The temperature program was the same as that of a previous study [44].

#### Lipid extraction and analysis

Lipids were extracted in accordance with an adaptation [45] of the method of Bligh and Dyer [43]. Lipids were extracted from lyophilized biomass with chloroform/methanol (2:1 v/v). The extracts were centrifuged at 5000 rpm for 25 min to completely dissolve the lipids in the organic phase. The organic phase was sucked out and washed twice by using the same volume of 0.15% (w/v) NaCl solution. Lipids were obtained after the purified chloroform layer was evaporated in a speed vacuum at 40 °C until a constant weight was achieved.

Lipid composition was determined through the gas chromatography (GC) analysis of fatty acid methyl esters (FAMEs). The method of FAME preparation and GC analysis are described in a previous study [17].

#### Confocal fluorescence microscopy analysis

Confocal fluorescence microscopy was carried out to illustrate the cell growth and lipid accumulation of *Y. lipolytica*. Approximately 10 μL of the cell suspension sample was collected from cultures at the stationary phase, subsequently stained with 10 μL of Nile red, and imaged with a laser scanning confocal fluorescence microscope (Leica TCS SP2) under an excitation wavelength of 543 nm.

## Data Availability

All data and material used in the current study are available from the corresponding author on reasonable request.

## Abbreviations

VFA: volatile fatty acids
DCW: dry cell weight
*Y*_X/S_: growth yield coefficient, g DCW/g VFAs
*Y*_L/S_: lipid yield coefficient, g lipid/g VFAs
TS: total solid
VS: volatile solid
rpm: rotations per minute
YPD: yeast peptone dextrose
FW: food waste
FVW: fruit and vegetable waste
N.D.: not detected
